# Renal Inadequacy

**Published:** 1880-04

**Authors:** 


### Selections.

---

### RENAL INADEQUACY.

Dr. Andrew Clark read an able paper before the Medical Society of London, at its meeting in Nov. 1879, "On Renal Inadequacy." He began by remarking that he was painfully struck by the great number of people suffering from ill health, of which no sufficient explanation could be given. Some of these cases took their rise in a feeble and disorderly nervous system, some in vicious digestion, some in an imperfectly acting skin, some in unsuitable conditions of life and work, some in abuse of tea, coffee, tobacco, alcohol, and other narcotics, and some in the derangement of the chemical changes which accompany and determine assimilation and deassimilation. But a far larger number were due to a deficient secretion of urinary solids. "By renal inadequacy, I mean that state of kidney in which it is unable, without material diminution of quantity, to produce a urine containing the average amount of solids, and of a specific gravity greater than 1014." The urine was pale, almost invariably free from albumen, and deposited no casts. He did not profess to determine what was the precise pathological state of the kidney; but he conjectured that it was one of slight withering and induration, just as sometimes the skin is found withered, hard, and incapable of producing a clear, unctuous sweat.

The symptoms and signs most commonly associated with renal inadequacy were flatulent dyspepsia, palpitation, with a very feeble and interrupted capillary circulation, a dry, shiny, waxy skin, numbness, tingling, cramps, and pains in the limbs, occasional flushes, worry of brain and general nervousness; sometimes, but rarely, evidences of gout. One knew in a given case that these sysmptoms were due to renal inadequacy, not merely because there was a grave deficiency in the excretion of urinary solids, but because whatever diminished that secretion or added to the amount of solids to be excreted, invariably in a short time, aggravated the patient's sufferings. Three things were of great importance in these subjects—they are exceedingly vulnerable; they repair very slowly the damage done by accident or disease; they bear very badly the shock, however slight, of surgical operations—a fact mentioned by Sir Francis Paget. As to prognosis, this state seemed capable of indefinite prolongation without serious injury to the organism. Under unfavorable circumstances and bad management, death might occur from some local imflammation, from cerebral or other hemorrhage, or from so-called pyæmic fever, springing unexpectedly out of some perhaps trifling operation. The special characters and appearances of patients who have been the subject of renal inadequacy for over four or five years are:—They have at least a marked and striking physiognomy; they increase in flesh; they become puffy without being distinctly œdematous; the skin becomes dried, more shiny and yellower; the features swollen almost to distension; the pupils are dilated; the lips and cheeks of a bluish red; the articulation deliberate and somewhat difficult and the whole intellectual tone and manner subdued and slow. From one side the physiognomy was like that of pernicious anæmia, from another like that of chronic Bright's disease, and yet it seemed distinct from both.

As to treatment much might be done by good management, by which he meant the adjusting of the quantity and quality of the food to the diminished excrementitious activity, the withholding of such agents as directly lessen the secretory power of the kidney, aiding the kidney in its work by making the supplementary excretory organs fulfill that part of the work which the kidney was unable to do; and generally by placing the patient in those conditions which would give the organism the greatest power for resisting the inroads of the disorder, and for making sufficient compensation when complete repair was unattainable. The tepid bath, followed by vigorous friction, the use of warm clothing, and the avoidance of passing exposure to cold and damp, with gentle exercise daily in the open air were indicated. The diet should be light; stimulants should be avoided except to the extent of one glass of claret or other light wine, twice a day. The medicines most useful were small doses of arsenic with reduced iron at meals, and an occasional mercurial alterative. If digestion is disturbed the iron and arsenic were discontinued, and bitters with alkalies between meals substituted, with a mercurial alterative every third night. He concluded by narrating a case which he first saw some years ago. By a strict adherence to a limited dietary, and by the use of purgatives and diaphoretics, this patient improved so much as to consider himself quite well; whereas, when he was taking food and wine every two hours, it seemed that the more he took the worse he became. A very remarkable fact about this case was that as his supplies of food and wine were reduced, the patient's urine steadily rose in density from 1003 up to a very fair standard, and in three weeks he left town declaring himself quite well. When seen six months ago this patient seemed and declared himself to be quite well, his only complaint being that he could not relax his dietary without being ill.

In the discussion which followed, Dr. C. T. Williams said these cases were generally treated as dyspeptics. He asked whether weight was gained or lost under the restricted diet; whether there was corpuscular deficiency or excess in the blood, or any signs of anæmia. Dr. Gilbart Smith asked whether it was due to renal defect or blood change. Did the kidneys refuse the blood or did the blood refuse to go to the kidneys? Had the organs been examined after death? Dr. Routh said there was no proof that the author's dictum was correct, and inclined to believe the ailment due to defective assimilation, and therefore lessened amount of salts in blood and urine rather than to renal inadequacy. Dr. Dowse had seen several cases similar to those described by Dr. Clark, but had never examined the kidneys after death. He did not for a moment doubt the existence of such a condition as renal inadequacy. Dr. Symes Thompson agreed that the kidneys must be at fault in these cases. He had not known that a diminished diet could increase the specific gravity of urine. Dr. Andrew Clark replied, urging the fact that proved the existence of such a state as renal inadequacy; that retention of excreta leads to disease, and that in a case he had at the London Hospital, nitrogenous diet increased the defective action of the kidneys. Some of the patients gained weight, others lost flesh on the strict regime. The blood did not appear abnormal. Apparently normal skin sometimes refused to perspire normally. Why should not a kidney which refused to act, yet show no apparent change?—*London Lancet, March, 1880.*

